# Editorial: Green biomanufacturing by industrial microorganisms: precise regulation and system optimization

**DOI:** 10.3389/fmicb.2026.2007772

**Published:** 2026-10-08

**Authors:** Xiang Zhou, Hongzhen Luo, Jun Liu, Shuo Han

**Affiliations:** 1 Institute of Modern Physics, Chinese Academy of Sciences, Lanzhou, China; 2 School of Life Science and Food Engineering, Huai'an University, Huai'an, China; 3 College of Food Science and Engineering, Central South University of Forestry and Technology, Changsha, China; 4 Division of Biological and Biomedical Systems, School of Science and Engineering, University of Missouri-Kansas City, Kansas City, MO, United States

**Keywords:** AI-driven bioprocess control, green biomanufacturing, industrial microorganisms, precise regulation, system optimization

The global pursuit of carbon neutrality and the transition toward a bio-based economy have placed industrial microbiology at the center of sustainable manufacturing. Yet the translation of laboratory-scale microbial innovations into robust industrial processes remains constrained by the inherent limitations of static control strategies, which are poorly suited to the dynamic and heterogeneous environments of large-scale biomanufacturing. Addressing these bottlenecks requires not only novel bioengineering tools but also a conceptual shift toward spatiotemporal metabolic control, dynamic feedback, and multi-scale system integration. Against this backdrop, this Research Topic brings together eight articles that approach green biomanufacturing from complementary perspectives, with coverage of precise molecular regulation, intelligent process control, and holistic system optimization.

A first theme concerns the design of green catalytic systems that improve the physicochemical properties of high-value bioactive compounds. The contribution by the group exemplifies this direction through the whole-cell-catalyzed cinnamoylation of phillyrin (Yang et al.). By combining *Aspergillus oryzae* whole cells with hydrophobic natural deep eutectic solvents (NADESs) in co-solvent systems, the authors achieved conversion rates of 97.32–99.00% and excellent 6′-regioselectivity (>99%) under optimized conditions. Importantly, the biocatalysts retained 80.09–97.95% of their original activity after six consecutive cycles. This work underscores how the rational pairing of biocatalysts with sustainable reaction media can expand the chemical space accessible to biotransformation. Future research could extend this system to bioreactor scale-up, examine NADES recycling, and elucidate the molecular basis of membrane permeability changes.

A second theme addresses the stability and productivity of industrial enzymes. The study on ancestral alginate lyase engineering provides a compelling example (Mu et al.). Starting from a *Flavobacterium* sp. enzyme heterologously expressed in *Escherichia coli*, the authors applied ancestral sequence inference to generate variants AncAlyA1 and AncAlyA2, which exhibited 132.4% and 87.3% increases in catalytic activity, together with markedly improved thermal and pH stability. AncAlyA2 retained approximately 60% of its activity under extreme conditions. Coupled with fed-batch fermentation strategy, enzyme activities of 5,164.8 U/mL and 4,220.1 U/mL were achieved. This study illustrates how evolutionary reconstruction, when integrated with process-level feeding optimization, can deliver biocatalysts that meet industrial performance criteria.

The third contribution shifts the focus from biocatalyst design to intelligent process control. To overcome overflow metabolism and the heavy reliance on manual intervention in high-density *E. coli* fermentation, the authors developed an artificial neural network (ANN)-based autonomous control system, NeuroStat-Ctrl (Zhou et al.). Using superfolder green fluorescent protein as a reporter, they first optimized DO-stat feeding and induction, achieving a 52.85% increase in specific fluorescence intensity. The ANN model recognized dissolved oxygen baselines in real time (*R*^2^ = 0.998) and was integrated with feeding control logic to enable fully autonomous operation. By mitigating feeding deviations inherent in fixed-threshold strategies and preventing metabolic overflow, this system offers a standardized and intelligent solution for high-throughput strain screening and parallel bioreactor validation.

Microbial consortia represent an avenue for enhancing carbon efficiency and product diversity. The study on engineered *Clostridium acetobutylicum* and *Clostridium ljungdahlii* syntrophic cocultures tackles the intrinsic carbon loss of glycolysis (Otten et al.). By transforming *C. acetobutylicum* with a synthetic acetone pathway and coculturing it with *C. ljungdahlii* to capture CO_2_ and H_2_, the authors achieved 246 mM isopropanol and 148 mM butanol within 64 h. Total product yields reached 84.7% on C-mol basis, compared with 65.6% for the monoculture. This work demonstrates that rationally designed syntrophic interactions can substantially improve carbon conservation and target chemical production.

A fifth theme concerns the valorization of renewable lignocellulosic feedstocks. The integrated strategy combining ternary DES pre-treatment with clostridial fermentation addresses the high cost of corn-/sugarcane-based butanol production (Shao et al.). Using a DES composed of choline chloride, succinic acid, and glycerol with water as co-solvent, the authors achieved 61.7% xylan removal and 93.6% glucan recovery from reed straw, with glucose yields reaching 87.9% after enzymatic hydrolysis. Fermentation of the real hydrolysate by *C. acetobutylicum* ATCC 824 produced ~6.5 g/L butanol. Notably, hydrochar prepared from enzymatic residues enhanced butanol titer to 11.5 g/L in synthetic medium containing typical inhibitors. This integrated pre-treatment, hydrolysis, fermentation, residue valorization chain is a model for complete lignocellulose use. Further work could focus on DES recycling and mitigating inhibition in real hydrolysates to close the titer gap with synthetic media.

The sixth and seventh contributions highlight the power of combining traditional parameter optimization with intelligent modeling and strain improvement for high-value natural products. The wild-type *Phaffia rhodozyma* GDMCC 2.218 was subjected to systematic fermentation optimization in 500 mL bioreactors, yielding 387.32 mg/L astaxanthin within 144 h, which improved to 400.62 mg/L upon scale-up to 5 L (Chen et al.). An LSTM prediction model achieved *R*^2^ = 0.978, confirming the feasibility of commercial-scale natural astaxanthin production. In parallel, the red yeast rice study employed combinatorial mutagenesis integrating atmospheric room-temperature plasma and heavy-ion radiation to obtain *Monascus purpureus* CSUFT-1, which showed a 1.67-fold increase in monacolin K production (Wu et al.). Through refinement of solid-state fermentation parameters, MK yield reached 32.71 mg/g, with scale-up stabilizing at approximately 24.66 mg/g. Together, these studies demonstrate that non-GMO and mutagenesis-based approaches, when coupled with precise bioprocess optimization and data-driven modeling, can achieve production levels competitive with engineered strains.

Finally, the eighth contribution extends the scope of green biomanufacturing to agricultural sustainability. Development of poly-ε-caprolactone nanospheres encapsulating *Smallanthus sonchifolius* essential oil achieved 98% encapsulation efficiency, significantly improved photostability, and provided sustained release (Wang et al.). The nanoencapsulated oil inhibited *Sclerotinia sclerotiorum* and *Valsa mali* by 93.6% and 56.9% *in vitro*, with *in vivo* inhibition rates of 94.45% and 92.5%, respectively. This eco-friendly strategy reduces reliance on chemical fungicides and exemplifies how biodegradable nanocarriers can bridge biomanufacturing with plant pathogen management.

Collectively, the Topic makes the field's open problems concrete. Scale-up validation remains shallow, so performance under industrial hydrodynamics and heterogeneity is untested. Sustainability claims are largely unquantified: titer, productivity, and yield are reported without carbon accounting, solvent recovery, or life-cycle metrics, leaving the “green” label qualitative. Data-driven models are trained on narrow, single-organism, single-product datasets, so their transferability across strains, products, and reactor scales is unresolved. Molecular regulation and process control are still optimized in isolation, and nano-enabled products face unresolved regulatory questions. Based on these gaps, we see three priorities: (1) closing the control loop across scales with hybrid mechanistic-AI models trained on multi-scale toward digital twins that co-design strain regulation and bioreactor dynamics; (2) making “green” measurable by adopting standardized TEA and LCA as co-equal optimization objectives; and (3) advancing predictive biocatalyst design. Pursuing the agenda would convert the advances collected in this Topic into scalable, verifiable industrial practice, and we hope that it stimulates the cross-disciplinary effort required to accelerate the transition from laboratory discovery to biologically driven industrial sustainability.

